# Circadian pain patterns in human pain conditions – A systematic review

**DOI:** 10.1111/papr.13149

**Published:** 2022-08-02

**Authors:** Nebojsa Nick Knezevic, Anthony Nader, Iulia Pirvulescu, Aby Pynadath, Behnoosh B. Rahavard, Kenneth D. Candido

**Affiliations:** ^1^ Department of Anesthesiology Advocate Illinois Masonic Medical Center Chicago Illinois USA; ^2^ Department of Anesthesiology University of Illinois Chicago Illinois USA; ^3^ Department of Surgery University of Illinois Chicago Illinois USA

**Keywords:** acute pain, analgesia, chronic pain, chronobiology, circadian rhythm

## Abstract

**Background:**

Chronobiology is the science of how physiological processes in the body follow a pattern of time. Pain has been shown to follow a circadian rhythm, with different types of pain having variable expression along this rhythm.

**Objective:**

This article reviews the nature of diurnal variations in pain along with a discussion of the mechanisms of circadian rhythm of pain.

**Evidence Review:**

We conducted a literature search on the PubMed and Google Scholar electronic databases, through April 2022. Publications were screened for English language, full‐text availability, and human subjects. Randomized controlled trials and observational trials were included. Data were extracted from studies on patients with acute or chronic pain phenotypes, which provide pain severity data and corresponding diurnal time points.

**Findings:**

The literature search led to the inclusion of 39 studies. A circadian pattern of pain was found to be present in nociceptive, neuropathic, central, and mixed pain states. Postoperative pain, fibromyalgia, trigeminal neuralgia, and migraines were associated with higher pain scores in the morning. Temporomandibular joint pain, neuropathic pain, labor pain, biliary colic, and cluster headaches increased throughout the day to reach a peak in the evening or night. Arthritis and cancer pain were not associated with any circadian rhythmicity. Furthermore, the circadian rhythm of pain was not found to be altered in patients on analgesics.

**Conclusion:**

The results of this review suggest that an understanding of diurnal variation may help improve therapeutic strategies in pain management, for instance through analgesic titration.

## INTRODUCTION

Chronobiology is the science that studies how physiological processes in the body follow a pattern of time. The suprachiasmatic nucleus (SCN) in the brain is considered to be the principal clock driving the human body.^1^ In fact, there are multiple clocks down to the molecular level, which work in tandem (via genes and feedback loops) with SCN to regulate body physiology.^1^ These involve hormone secretion, neuropeptides, and modulation of receptor expression. As a field, chronomedicine has been mentioned and discussed for the past few decades, yet it has only recently garnered interest as a specialty of its own.^2^

The circadian pattern of pain has been demonstrated in both animal and human studies.^3^, ^4^ Preclinical studies on this topic have mostly involved mice and rats exposed to heat stimuli or chemically‐induced inflammation, followed by the assessment of discomfort via behavioral tests. These preclinical studies have typically concluded that pain behaviors are increased at night, when the rodents are most active.^4^

Clinically, the chronobiology of pain has been poorly studied, especially in states of acute pain. This review aims to provide an updated compilation of circadian rhythms of pain described clinically in both acute and chronic pain conditions, as well as touch on known contributory mechanisms, in humans and rodents alike. A better understanding of the chronobiology of pain would enable using knowledge of this phenomenon to aid in the development of therapeutic strategies.

## METHODS

### Search strategy and data collection

We conducted a comprehensive bibliographic search on the PubMed and Google Scholar electronic databases, through April 2022. Additional records were identified and assessed from recently published reviews. Data extraction was focused on chronobiology in both acute and chronic pain conditions. The search was conducted using the following strategy: (pain) AND ((chrono‐) OR (circadian) OR (diurnal)). Publications were screened for English language, full‐text availability, and human subjects.

Authors A.N., I.P., A.P., and B.B.R. independently reviewed the titles, abstracts, and full texts according to the inclusion criteria. Consensus between authors was sought in case of discrepancies for the inclusion or exclusion of studies. Data from the resulting 39 publications were synthesized and presented in this review organized by type of pain.

### Inclusion and exclusion criteria

**FIGURE 1:**
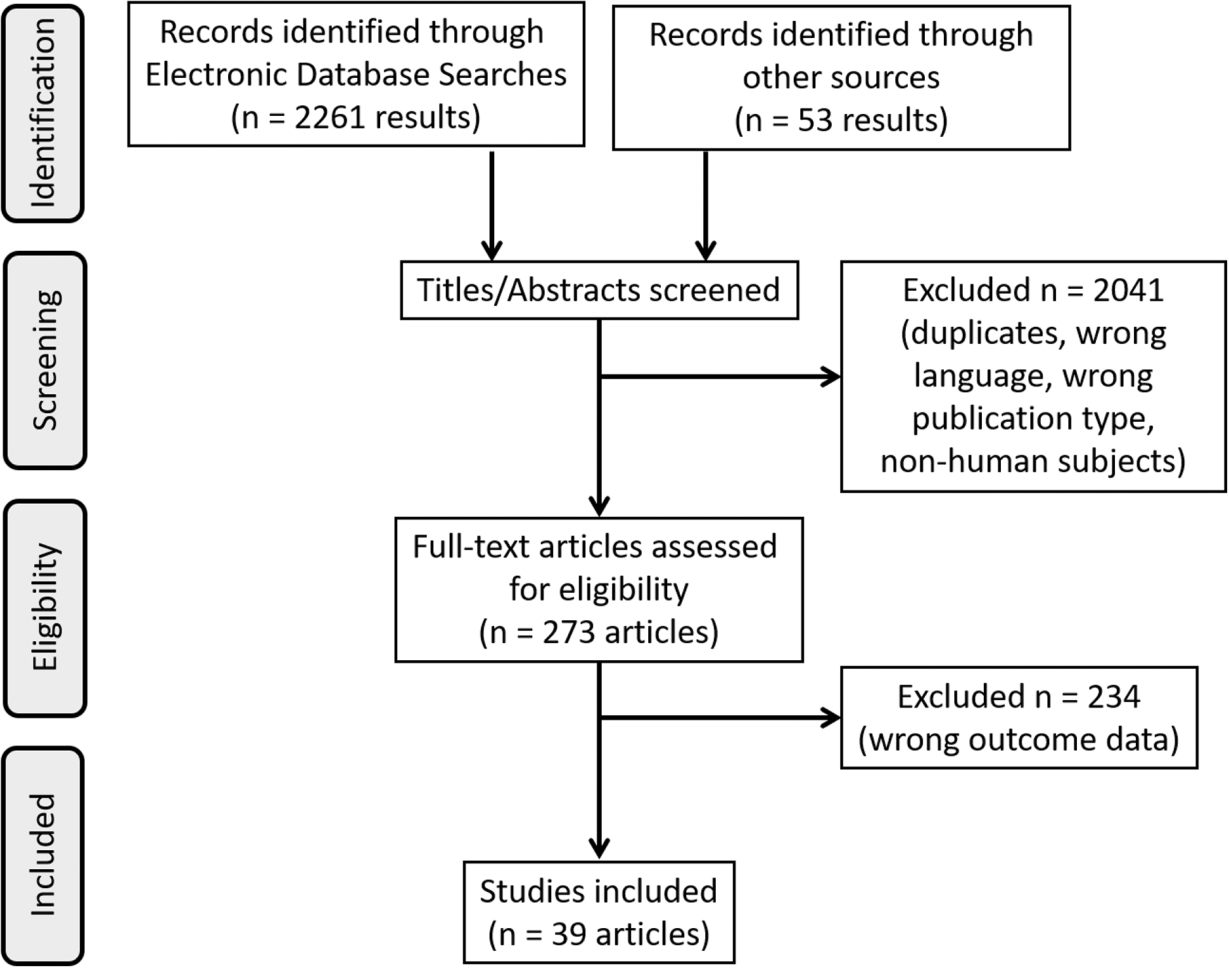
Flow diagram of literature search.

We included both randomized controlled trials and observational trials, including prospective and retrospective analyses of cohort, cross‐sectional and crossover study designs. Case reports and review articles were not included. Studies must include human patients with acute or chronic pain phenotypes. Our primary outcome was pain severity data and corresponding diurnal time point(s). The data extracted also included the study design and number of participants. The literature search and screening results are described in Figure 1.

### Risk of bias assessment

Authors A.N. and I.P. independently assessed the risk of bias using the Agency for Healthcare Research and Quality checklist.^5^ In the event of discrepancy, a consensus was reached with the assistance of author N.N.K. The checklist used provides clear criteria to assess risk of selection, performance, attrition, detection, and reporting biases. The criteria is composed of up to 16 yes/no questions, customized for study designs including randomized controlled trials (RCT), cohort and cross‐sectional trials. The results of the risk of bias assessment are presented in Table 1 and were used to inform quality of evidence analysis, when synthetizing the results.

**TABLE 1:** Risk of bias assessment of included studies where green denotes a low risk of bias, yellow indicates an unclear/medium risk of bias, and red symbolizes a high risk of bias.

Author	Selection bias	Performance bias	Attrition bias	Detection bias	Reporting bias
Aya et al.^6^					
Costa‐Martins et al.^7^					
Desai et al.^8^					
Debon et al.^9^					
Rigas et al.^10^					
Minoli et al.^11^					
Boscariol et al.^12^					
Song et al.^13^					
Kwon et al.^14^					
van Grootel et al.^15^					
Bellamy et al.^16^					
Caumo et al.^21^					
Bellamy et al.^26^					
Bellamy et al.^27^					
Zhang et al.^28^					
Allen et al.^29^					
Levi et al.^30^					
Odrcich et al.^49^					
Gilron et al.^50^					
Tomson et al.^51^					
Solomon^52^					
Fox and Davis^53^					
Alstadhaug et al.^54^					
Soriani et al.^55^					
de Tommaso et al.^56^					
Gori et al.^57^					
Packard et al.^58^					
Park et al.^59^					
van Oosterhout et al.^60^					
Kikuchi et al.^61^					
Rozen and Fishman^62^					
de Coo et al.^63^					
Lee et al.^64^					
Steinberg et al.^65^					
Saini et al.^67^					
Campagna et al.^68^					
Gagnon et al.^69^					
Currow et al.^70^					
Glynn and Lloyd^71^					

## RESULTS

### Clinical studies investigating the circadian nature of acute pain

**FIGURE 2:**
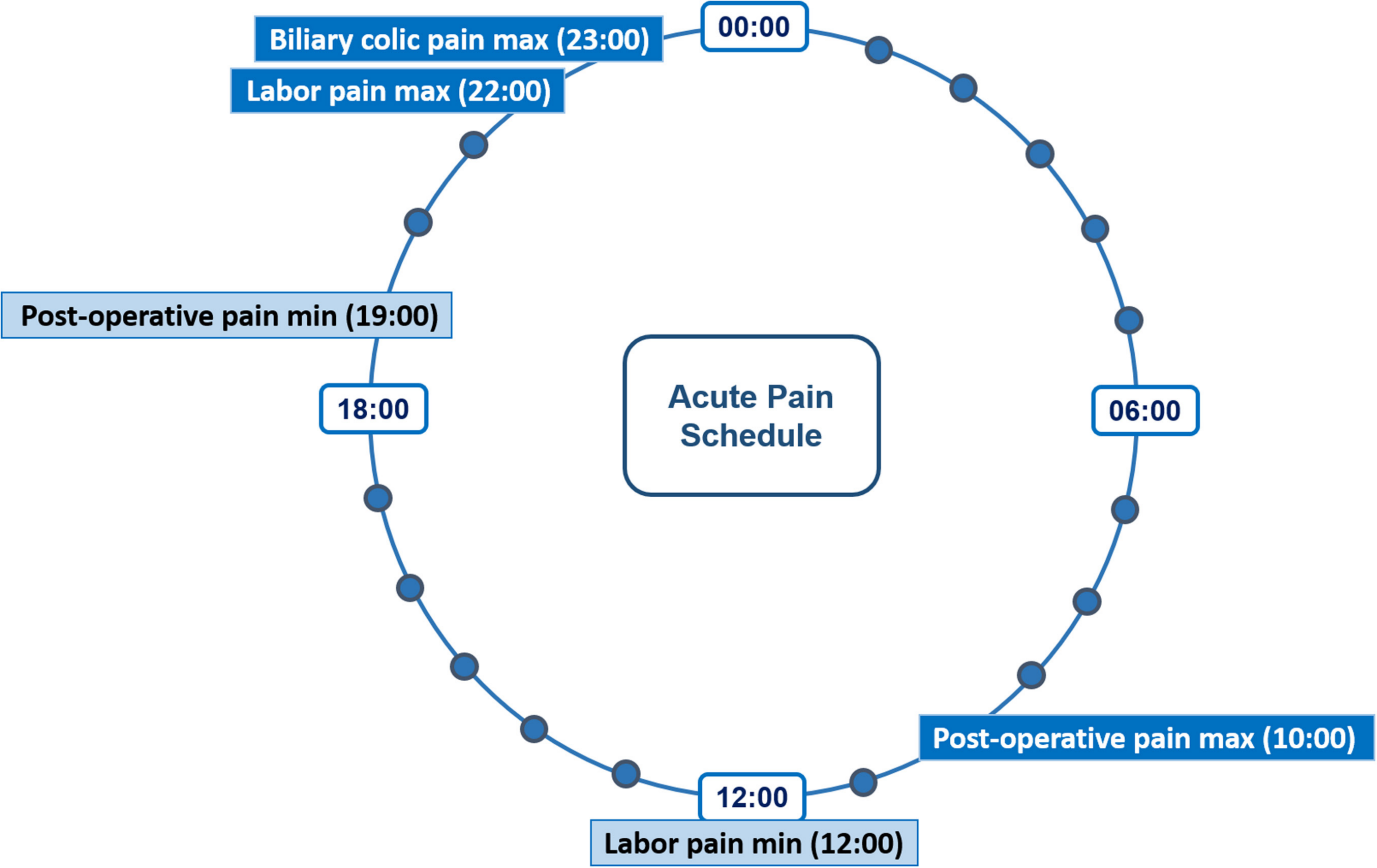
Chronobiology of acute pain.

A visual representation of the chronobiology of acute pain phenotypes discussed is seen in Figure 2, and a summary of the clinical studies is provided in Table 2.

**TABLE 2:** Circadian rhythms in different types of acute pain.

Author	Type of pain	Study design; Number of participants	Results
Aya et al.^6^	Labor pain	Observational cohort; 222 pregnant women	Women who experienced labor pain in the morning from 7:00 to 13:00 had an average visual analogue pain score of 75.6 when compared to 87.6 for women in evening.
Costa‐Martins et al.^7^	Labor pain	Prospective observational study; 81 pregnant women	Greater labor pain severity, longer latency phase of labor as well as shorter duration of analgesics efficacy at night.
Desai et al.^8^	Labor pain	Prospective cohort; 1657 pregnant women	Greater labor pain scores before epidural analgesia in evenings and nights than mornings (6.67 ± 2.5) and afternoons
Debon et al.^9^	Labor pain	Observational cohort; 194 pregnant women	No significant difference for labor pain assessed by VAS before epidural administration of ropivacaine between different circadian groups of patients. Longer duration of analgesia in the morning and afternoon groups than night and evening groups
Rigas et al.^10^	Biliary colic	Observational cohort; 50 participants	Pain follows a circadian periodicity with the peak pain at 00:25
Minoli et al.^11^	Biliary colic	Observational cohort; 54 participants	Pain was found to follow a circadian pattern with a peak occurring at 21:30
Boscariol et al.^12^	Postoperative: hysterectomy	Randomized, parallel, controlled; 103 patients	Day 1 postop, all types of pain were worst at 08:00 compared to 12:00, 16:00 and 20:00 Day 2 postop, morphine requirement was increased at 08:00 and 12:00 compared to 16:00 and 20:00
Song et al.^13^	Postoperative: abdominal surgery	Randomized controlled; 84 patients	Morning operations (8:00–12:00) required a higher dose of anesthetic drugs than evening operations (18:00–22:00)
Kwon et al.^14^	Postoperative: hip surgery	Randomized controlled; 44 patients	No significant difference between groups in terms of pain scores at any time point

#### Labor pain

Women with labor pain were found to experience more pain in the morning than in the evening. A cohort study with 222 first‐time pregnant women carrying singleton vertex pregnancy, spontaneous onset labor, cervical dilatation (3–5 cm), ruptured membranes, and normal fetal heart rate tracings were grouped into four different time periods and asked to rate their pain on a visual analogue scale (VAS) at the time of referral to anesthetist for epidural anesthesia. A circadian rhythm was observed with pain in the morning being less than in the afternoon and evening. Women who experienced labor pain in the morning from 07:00 to 13:00 had an average VAS of 75.6 when compared to 87.6 for women in evening (*p* < 0.0001).^6^

In a similar prospective observational study done on 81 pregnant women receiving patient controlled epidural analgesia (PCEA), the intensity of pain in the early stage of labor (3 cm of cervical dilatation) before receiving PCEA was significantly higher at night. It was also observed that the latency period was longer during the night and the duration of the pharmacologic effect was longer during the day. When pharmacological effects and analgesic consumption during labor pain were assessed via VAS, labor pain was significantly greater during the night than daytime. Additionally, longer latency periods of labor as well as shorter duration of pharmacological effect of analgesics at night were reported.^7^

A very large cohort study including 1657 pregnant women showed significantly greater (*p* < 0.001) levels of labor pain before epidural analgesia in evenings (6.95 ± 2.4) and nights (7.38 ± 2.2) than mornings (6.67 ± 2.5) and afternoons (6.49 ± 2.7).^8^ Debon et al. conducted a cohort study including 194 pregnant women. When epidural administration of ropivacaine was completed on different circadian groups, no significant difference on the VAS was reported. There was a longer duration of analgesia in patients included in the morning group (110 ± 25 min) and in the afternoon group (117 ± 23 min) compared with the patients included in the night group (94 ± 23 min) and in the evening group (91 ± 23 min) (*p* < 0.01).^9^

In summary, labor pain appears to be highest between evening and night, and lowest between morning and afternoon.

#### Biliary colic

Fifty patients who underwent emergency or elective cholecystectomies for symptomatic biliary tract stones were interviewed in their first postoperative week for variables such as time of pain onset, location, duration, and number of pain episodes. Variables were collected to describe pain episodes both pre‐ and post‐surgery. The pain was found to follow a circadian periodicity (*p* = 0.0032) with the peak pain at 00:25. Only 40% of study subjects reported that the pain was related to a meal, thus it is not exclusively related to the effect of the evening meal causing a late‐night peak in the biliary colic.^10^

In a similar study, ultrasonography was used to study and document the onset time of biliary pain in 54 patients with cholelithiasis. Pain was found to follow a circadian pattern with a peak occurring at 21:30. Higher pressures in the sphincter of Oddi during the day have also been speculated as a reason for this circadian variation.^11^ It should be noted that this study exhibited a risk of performance bias.

In summary, biliary colic pain appears to be the highest between 21:30 and 00:25.

#### Circadian variation in postoperative pain

There has also been a considerable interest in the variation of analgesic requirements in postoperative pain. A temporal variation in postoperative pain was described among women who underwent hysterectomies, in a low risk of bias RCT including 103 patients. Different types of pain were studied including rest pain, pain evoked by sitting, pain by forced expiration, and pain by coughing. On postoperative day 1, all types of pain were at a maximal intensity at 08:00 when compared to the pain experienced at 12:00, 16:00 and 20:00 (*p* < 0.05). A similar analysis for morphine requirements was significant for increase in morphine consumption on postoperative day two at 08:00 and 12:00 when compared to that at 16:00 and 20:00 (*p* < 0.05). However, no significant variation in morphine requirement was found on postoperative day one.^12^

A low risk of bias RCT including 84 patients who underwent abdominal surgery found that morning operations (8:00–12:00) required a higher dose of anesthetic drugs than evening operations (18:00–22:00). Additionally, evening surgeries were associated with more sleep disruptions, which may increase postoperative pain perception.^13^

A prospective, randomized clinical trial was conducted to compare postoperative outcomes in 44 patients who underwent either morning surgery, characterized by high cortisol levels, or afternoon surgery, characterized by low cortisol levels. The results of the study showed no significant difference between groups in terms of pain scores at any time point. Compared to morning surgery, however, afternoon surgery allowed for a more rapid recovery of cortisol levels to baseline, and lower IL‐6 and Il‐8 levels in the first two postoperative days.^14^

In summary, postoperative pain appears to be highest in the morning and lowest in the evening.

### Clinical studies investigating the circadian nature of chronic pain

**FIGURE 3:**
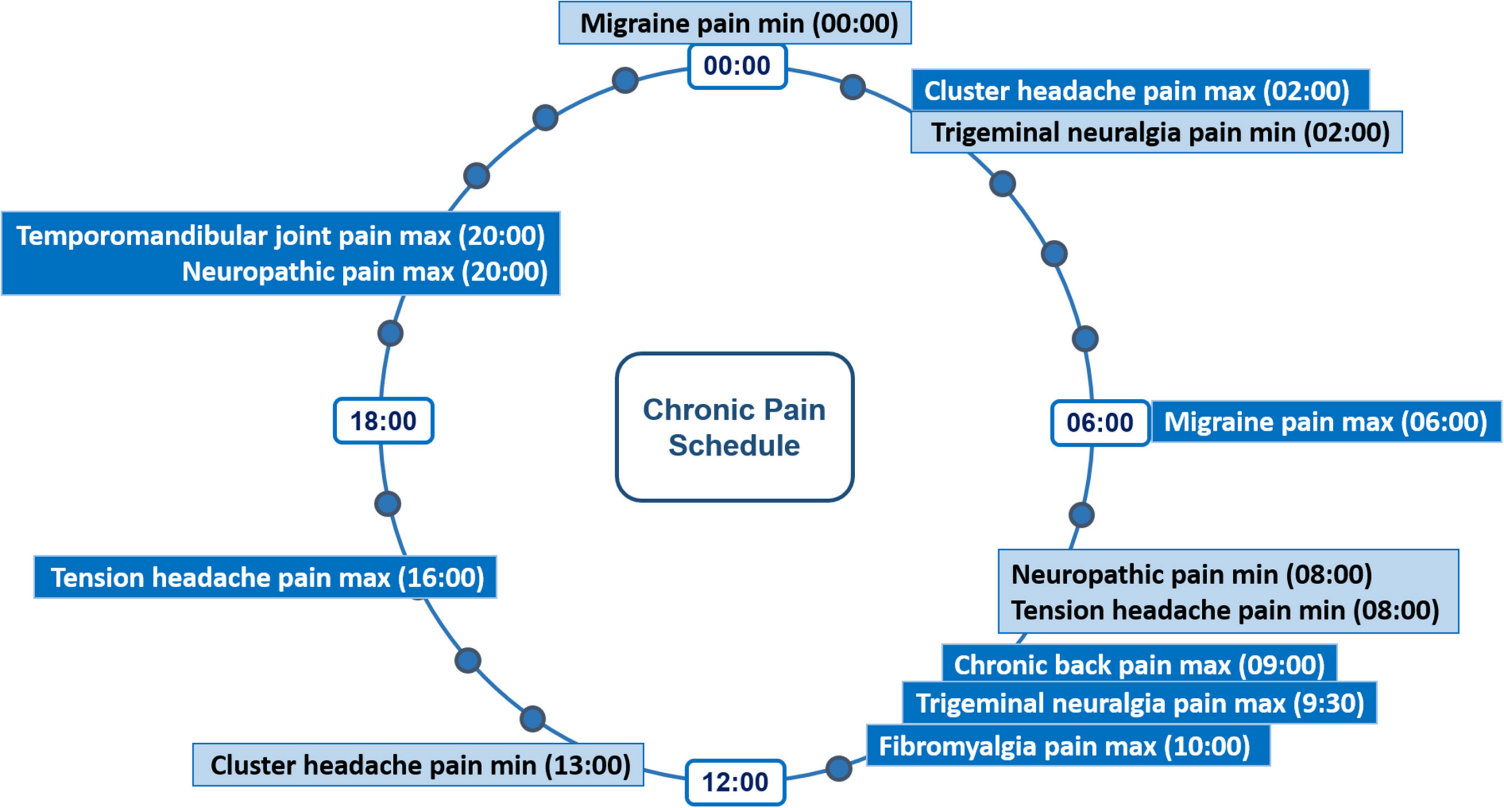
Chronobiology of chronic pain.

A visual representation of the chronobiology of chronic pain phenotypes discussed is seen in Figure 3, and a summary of the clinical studies is provided in Table 3.

**TABLE 3:** Circadian rhythms in different types of chronic pain.

Author	Type of pain	Study design; Number of participants	Results
van Grootel et al.^15^	Temporomandibular joint pain	Randomized controlled; 133 patients	TMJ pain intensity was highest late in the day (before dinner or bedtime)
Bellamy et al.^16^	Fibromyalgia	Observational cohort; 21 female patients	Pain more severe in the morning compared to afternoon
Caumo et al.^21^	Fibromyalgia	Cross‐sectional; 18 patients	Negative correlation between pain pressure threshold and aMT6s levels between 6:00–18:00, when fibromyalgia patients' aMT6s is higher than control
Bellamy et al.^26^	Rheumatoid arthritis	Cross‐sectional; 13 patients	Lowest pain level occurred around 17:00
Bellamy et al.^27^	Hand osteoarthritis	Observational cohort; 21 patients	Pain severity least at 16:10
Zhang et al.^28^	Knee osteoarthritis	Observational cohort; 241 patients	Lower pain intensity in the afternoon
Allen et al.^29^	Hand, hip or knee osteoarthritis	Observational cohort; 157 patients (hand (40), hip (32), and knee (85))	Pain severity increased during the morning and early afternoon, and declined during the evening.
Levi et al.^30^	Hip or knee osteoarthritis	Double‐blind, crossover; 66 patients	Some circadian profiles were unimodal (*n* = 30), with a single peak between 8 a.m. and 2 p.m. (*n* = 3), between 2 and 8 p.m. (*n* = 19), or between 8 p.m. and 8 a.m. (*n* = 8). Other profiles were bimodal, with both a morning and an evening peak (*n* = 23). In four series, self‐rated pain intensity varied little throughout the day.
Odrcich et al.^49^	Neuropathic pain	Randomized, double‐dummy, crossover; 85 patients	Pain worsened throughout the day from 08:00 to 20:00 pattern maintained even with analgesics
Gilron et al.^50^	Neuropathic pain	Randomized, double‐dummy, crossover; 56 patients	Pain score increased throughout the day from 08:00 to 20:00, during the pretrial baseline and also during the treatment with the drugs
Tomson et al.^51^	Trigeminal neuralgia	Observational cohort; seven patients taking carbamazepine	The frequency of pain attacks was lowest during night hours (23:00–5:00), and highest during the morning (8:00–11:00).
Solomon^52^	Migraine headache	Prospective cohort; 15 patients	Onset of migraine was greatest from 6:00–12:00
Fox and Davis^53^	Migraine headache	Prospective cohort; 1698 patients	Migraine attacks peaked from 4:00–9:00
Alstadhaug et al.^54^	Migraine headache	Retrospective cohort; 58 female patients	Pain was found to peak at 13:40
Soriani et al.^55^	Migraine headache	Prospective cohort; 115 children patients	First peak of in the afternoon (16:48) and second peak in the early morning (06:35)
de Tommaso et al.^56^	Migraine headache	Observational cohort; 786 patients	The frequency of migraine attacks is higher throughout the day, with peaks at 10:00 and 22:00. Attacks were less frequently noted at night (3:00).
Gori et al.^57^	Migraine headache	Observational cohort; 100 patients	42% of patients experience more than 75% of their attacks at night and in the early morning hours (3:00–7:00).
Packard et al.^58^	Migraine headache	Randomized controlled; 61 patients	No difference in mean pain level between the morning and the afternoon.
Park et al.^59^	Migraine headache	Observational cohort; 82 patients	Migrainous headache characteristics presented most frequently at 06:00–12:00, and least frequently at 18:00–00:00 and 00:00–06:00. The same pattern was seen for the occurrence of all headache types (migraine and non‐migraine).
van Oosterhout et al.^60^	Migraine headache	Observational cross‐sectional; 2389 patients	Migraine attacks most often began at 04:00–06:00 (15.4% of total) or 06:00–08:00 (11.8% of total).
Kikuchi et al.^61^	Tension headache	Prospective cohort; 31 patients	Intensity of tension headaches was significantly lower in the morning and the peak was in the late afternoon (16:00)
Rozen and Fishman^62^	Cluster headache	Observational, survey‐based; 1134 patients	Peak between 00:00 and 03:00, most commonly 02:00
de Coo et al.^63^	Cluster headache	Cross‐sectional; 147 headache patients	Attacks occurred most often between 00:00 and 4:00 and least often between 12:00 and 16:00
Lee et al.^64^	Cluster headache	Multicenter, prospective cohort; 175 patients	Nighttime attacks were predominant early in the disease course, while daytime attacks increased with disease progression and decreased in patients with the most advanced disease course
Steinberg et al.^65^	Cluster headache	Observational cohort; 475 patients (episodic (421) and chronic (54))	The most commonly reported time interval for attack was nighttime (2:00–4:00), and the lowest rates were noted around late morning and early afternoon (10:00–14:00). A third of patients noted no rhythmicity.
Saini et al.^67^	Breakthrough pain in cancer	Prospective cohort; 123 patients	More breakthrough pain in the morning hours when compared to evening, with a peak between 9:45–10:30
Campagna et al.^68^	Cancer Pain	Prospective longitudinal cohort; 92 patients	Acrophase between 12:15–12:30
Gagnon et al.^69^	Breakthrough pain in cancer patients	Retrospective analysis of prospective cohort study; 104 patients	Patients without delirium needed more analgesia in the morning and patients with delirium required more analgesia in the evening and at night
Currow et al.^70^	Cancer pain	Randomized, double‐blind, crossover, placebo‐controlled; 42 patients	No significant difference between the level of pain for patients on morphine in the morning versus in the afternoon
Glynn and Lloyd^71^	Cancer Pain	Prospective cohort; 41 patients	Pain increases throughout day and reaches maximum at last time point (22:00)

#### Temporomandibular joint pain

In a study conducted among 133 patients with temporomandibular disorders in a 2‐week period, patients recorded pain on a VAS. For most patients (79%), pain intensity was found to be highest late in the day (before dinner or bedtime). For the rest of the patients (21%), pain was found to be highest early in the day.^15^

#### Fibromyalgia

In fibromyalgia (FM), pain was found to be most severe in the morning compared to the afternoon. In a study of 21 female patients with FM, subjects were asked to document their pain on a VAS at 6 specified time points for 10 consecutive days. Data were analyzed for 24‐h and 7‐day time effects, and for diurnal and weekly rhythms using the cosinor technique.^16^ This technique is a statistical method for biologic rhythm analysis. Acrophase is the time when peak of the rhythm occurs and bathyphase is the time when the lowest part of the rhythm occurs.^17^ For patients with low dolorimetry scores (<2.25 kg), a diurnal variation in pain was observed, with maximum onset of pain in the morning when compared to the afternoon. However, no diurnal variation in pain was observed in patients with a high dolorimetry score (>2.25 kg).^16^ A dolorimeter is a pressure algometer. Examiners placed the rubber tip on the examination site and gradually increased the pressure at a rate of approximately 1 kg/cm^2^ per second. The patient was asked to report the moment when the sensation at the examination site changed from that of pressure to that of pain. The reported dolorimetry score is the mean of the sites examined.^18^ Dolorimetry scores show a wide variation in the population, with median values for women being 4.25 and 6.0 kg/cm^2^ for men.^19^, ^20^ In a population survey, the mean dolorimetry score in persons with FM was approximately 2.7 kg/cm^2^.^20^

In a 2019 study, aMT6s (a melatonin indicator found in urine) secretion levels in 18 patients with FM were compared to healthy patients using the pain pressure threshold as the primary outcome.^21^ Patients verbally reported their perceptions of pain when an algometer was applied to tender points on the body. Urine samples were collected every 6 h during a 24 h period. A general estimating equation model showed statistical significance (*p* = 0.001) in aMT6s levels over the 24‐h period confirming a disruption in circadian rhythmic secretion of aMT6s. During daytime hours (06:00–18:00), FM subjects secreted 41.54% of their daily aMT6s secretion load while the healthy control group only secreted 20.73%. Higher secretion levels during night periods (24:00–6:00) correlated to overall lower depressive symptoms. Higher secretion during the hours of 6:00–18:00 correlated to more trigger points and a lower quality of sleep. A multivariate linear regression model was created using pain pressure threshold measurements as the dependent variable. Using the Bonferroni test, a negative correlation between pain pressure threshold and aMT6s levels was observed between 6:00 and 18:00. During the same time interval, a positive correlation between the number of trigger points and aMT6s levels was found.^21^

Fibromyalgia and major depression disorder (MDD) have common symptoms and markers in the hypothalamic–pituitary–adrenal axis and autonomic dysregulation systems (both help regulate stress).^22^, ^23^, ^24^ The same study also compared a third group with MDD to the healthy group and found that during daytime hours (6:00–18:00), MDD subjects secreted 60.71% of their daily aMT6s secretion load while the healthy control group only secreted 20.73%. Showing a positive correlation with scores from the Hamilton Depression Rating Scale (assessments done pre‐clinically), both subjects with FM and MDD showed higher levels of aMT6s secretion during the 06:00–18:00 time interval (*ρ*: 0.49, *p* = 0.03; *ρ*: 0.67, *p* = 0.001).^21^

In summary, fibromylagia pain appears to be highest in the morning.

#### Arthritis

The circadian rhythm of pain was found to be different in rheumatoid arthritis (RA) when compared to osteoarthritis (OA). RA patients had a peak onset of their pain in the morning, whereas the pain from OA got worse as the day progressed.^25^ Bellamy et al. studied the variation of pain in 13 patients with RA. Patients were asked to rate their pain on a VAS six times a day. It was found that the bathyphase (lowest point of a mathematical model fitted to a time series describing a rhythm) of pain during the day occurred around 17:00 (*p* < 0.05).^26^ In a different study, 21 patients with hand OA were asked to record their pain six times per day for 10 consecutive days. A circadian rhythm was identified in the severity of pain (*p* = 0.013). Pain was found to have a bathyphase value of at least 16:10.^27^

In a large and low risk of bias clinical trial, Zhang et al. studied circadian rhythm of knee pain in 241 patients with knee OA in different testing sessions including 8:00–10:00, 10:00–12:00, 13:00–15:00 and 15:00–17:00. Based on VAS, patients in this study showed lower levels of pain intensity in the early afternoon (13:00–15:00) compared to the early morning (08:00–10:00, *p* < 0.005) and late morning (10:00–12:00, *p* < 0.001). Furthermore, patients showed lower levels of pain intensity in the late afternoon than the late morning (*p* = 0.22).^28^

In a cohort study, including 157 patients with osteoarthritis of the hand, hip, or knee, Allen et al.^29^ found that pain severity was increased during the morning and early afternoon, and pain declined during the evening.

Levi et al performed a double‐blind, crossover trial on 66 patients with hip or knee osteoarthritis. About half of the patients, a unimodal circadian profile was noted, with a single peak between 8 a.m. and 2 p.m. (*n* = 3), between 2 and 8 p.m. (*n* = 19), or between 8 p.m. and 8 a.m. (*n* = 8). In 23 patients, a bimodal circadian profile was seen, with both a morning and an evening peak. In 4 of the 66 patients, self‐rated pain intensity varied little throughout the day.^30^

It is suggested by Petrovsky and colleagues that in RA, patients' cortisol plays an anti‐inflammatory role by reducing endogenous proinflammatory cytokine levels, while melatonin plays a proinflammatory role by inhibiting an enzyme (matrix metalloproteinase‐9) that causes joint damage and inflammatory pain.^31^, ^32^, ^33^ The SCN, located in the hypothalamus, helps regulate circadian rhythmic secretions of hormones, cytokines, chemokines, as much of the immune system. Hence, during sickness, an issue with the SCN could affect the neuroimmune responses and illicit an unwanted response to inflammation.^34^

Rheumatoid arthritis usually results in worse symptoms in the morning^35^, ^36^, ^37^ and lower pain intensities in the late afternoon.^26^, ^27^, ^38^ A link to pain and secretion of cortisol^39^, ^40^ and melatonin^41^, ^42^, ^43^ has been found. However, which peak of secretion causes the heightened morning pain in RA patients still shows some conflicting reports. Regarding cortisol, studies showed that plasma cortisol concentrations and circulating cortisol levels decrease at night and peak in the morning.^39^, ^40^, ^44^ Regarding melatonin, one group demonstrated that when compared to healthy subjects, RA patients had significantly increased melatonin levels at midnight.^45^ Another study reported that when compared to placebo‐treated subjects, melatonin‐treated subjects had an increase in inflammatory indicators.^34^, ^46^

A 2019 study by Poolman et al.^47^ showed that RA increases circadian rhythmicity through chronic inflammation, changing expressions/pathways for peripheral blood mononuclear leukocytes (PBML), phospho‐STAT3/ATF2, and hepatic ceramide synthases. Subjects 16–80 years of age (*n* = 10) used an actigraph and sleep diary at home for 1 week, followed by a 24 h stay at the clinical research facility. Non‐circadian conditions were set for standardized neutral light, entertainment, nutrition, and prescribed inactivity. PBML, saliva, and serum were collected at 06:00 and 18:00.^48^ In the healthy control group, 25 genes were expressed differently from the morning (06:00) to the evening sample (18:00) compared to 104 in subjects with RA. When the hypothalamic–pituitary–adrenal (HPA) axis was tested using non‐parametric analytical approaches, the subjects with RA showed a strong correlation between core clock genes and their PBML. The same two groups were also tested for diurnal gene expression, diurnal proteome, and immune cell responses. The RA subjects in most cases showed rhythmic gains. The group also used a collagen‐induced arthritis mouse model and found that the rhythmic trends of RA were consistent across most cases of inflammatory arthritis.^47^

To summarize, arthritis pain does not show a clear association with a circadian pattern. It is frequently bimodal, and one or more peaks may occur throughout the daytime.

#### Neuropathic pain

Neuropathic pain such as diabetic neuropathy (DN) and postherpetic neuralgia (PHN) was also shown to exhibit a diurnal variation of pain. In a double‐dummy (“double‐dummy” design is a form of double‐blind study which allows additional insurance against bias and all patients are given both placebo and active doses in alternating periods), 4‐period, crossover comparison study, 85 patients were randomized and then assigned to one of the predetermined sequences which included treatment with a placebo, gabapentin, morphine, and morphine‐gabapentin combination. Each treatment period was 5 weeks long including 3 weeks of dose titration, 1 week at maximum tolerated dose and 1 week of tapering and washout. Patients were asked to rate their pain on a numerical rating scale (NRS) at 08:00, 16:00, and 20:00, from 7 days before the study until the end of the study. The circadian nature of the pain was analyzed from the baseline data which patients recorded 7 days prior to beginning the study. Thirty‐four out of 55 patients having DN and 26 out of 30 having PHN showed a temporal relationship, with pain worsening throughout the day from 08:00 to 20:00 (*p* < 0.0001). The pain scores were again analyzed when patients were on analgesics, and it was noticed that the diurnal variation of pain was maintained even when patients were treated with analgesics (*p* < 0.0001). In patients undergoing treatment, two‐way measures by analysis of variance (ANOVA) revealed a significant time of day (*p* < 0.001) and a significant effect of treatment (*p* = 0.004), but there was no interaction between the two (*p* = 0.38).^49^ It should be noted that this study exhibited a risk of performance bias.

Gilron et al. analyzed data from a double‐dummy, 3‐period crossover trial where 56 patients (DN = 40, PHN = 16) were randomized to receive treatment with gabapentin, nortriptyline and a gabapentin‐nortriptyline combination in a predetermined sequence. Patients recorded their pain on a numerical scale at 08:00, 16:00 and 20:00, 7 days before starting the study and continued the same until the end of study. The treatment data came from 47 patients. It was found that the pain score increased throughout the day, during the pretrial baseline and also during the treatment with the drugs.^50^ It should be noted that this study exhibited a risk of reporting bias.

Tomson et al. performed a cohort study including seven patients with idiopathic trigeminal neuralgia, a form of neuropathic pain affecting the trigeminal nerve in the face. Of note, all patients included were receving carbamazepine treatment. The study found that the frequency of pain attacks was lowest during night hours (23:00–5:00) and highest during the morning (8:00–11:00).^51^

In summary, neuropathic pain appears to be highest in the evening, and lowest in the morning. Trigeminal neuralgia shows a different pattern, with the highest pain levels in the morning, and lowest at night.

#### Headache

##### Migraine headache

In a low risk of bias cohort study of 15 patients with migraines taking place over 20 weeks, a total of 211 migraines were documented. The incidence of migraine onset from 06:00 to 12:00 was 3.3 times greater than the incidence during other 6 h periods (*p* < 0.05).^52^ A much larger study, including 1698 patients who experienced 3582 migraine attacks, found that 48% of migraine attacks occurring from 04:00 to 09:00. For the remaining time, the rate was 115 ± 23 attacks per hour (*p* < 0.001).^53^ It should be noted that this study exhibited a risk of detection bias.

A study on female patients, including 58 patients prospectively recorded migraine attacks for 12 consecutive months with a total of 2314 attacks. The pain was found to peak around 13:40 and the peak/low ratio was 25.6 (95% CI:8.3–78.6).^54^ A study on children assessed 115 patients aged 5–18 years, and showed a peak in the afternoon (16:48, *p* < 0.001) and the second peak in the early morning (06:35, *p* = 0.002).^55^ It should be noted that this study exhibited a risk of performance bias.

A large cohort study on 786 migraine patients found that the frequency of migraine attacks is higher throughout the day, with peaks at 10:00 and 22:00. Attacks were less frequently noted at night (3:00).^56^ Another cohort study, including 100 patients, determined that 42% of patients experience more than 75% of their attacks at night and in the early morning hours (3:00–7:00).^57^ It should be noted that this study exhibited a risk of selection bias.

A more recent RCT on 61 migraine patients did not identify differences in mean pain levels between morning and afternoon.^58^ A cohort study on 82 migraine patients found that migrainous headache characteristics presented most frequently at 06:00–12:00, and least frequently at 18:00–00:00 and 00:00–06:00. Of note, the same pattern was seen for the occurrence of all headache types (migraine and non‐migraine).^59^ Finally, in a very large cross‐sectional study including 2389 patients, van Oosterhout et al.^60^ identified that migraine attacks most often began at 04:00–06:00 (15.4% of total) or 06:00–08:00 (11.8% of total).

In summary, migraine pain and frequency appears to be highest in the morning, and lowest between between late evening and nighttime.

##### Tension headache

Thirty‐one patients were assessed for tension headaches using a computerized ecological momentary assessment. It was found that the intensity of tension headaches was significantly lower in the morning and the peak was in the late afternoon (16:00, *p* = 0.0005).^61^

##### Cluster headache

In a study with 1134 patients, 58% of patients had attacks between 19:00 and 07:00, with the majority of patients showing a peak between 24:00 and 03:00, and 41% of responders showing a peak at 02:00 (*p* < 0.00001).^62^ It should be noted that this study exhibited a risk of selection bias.

A cross‐sectional study assessed sleep and chronotype in a cohort of 147 episodic and chronic cluster headache patients and non‐headache controls. Results showed that cluster headache attacks occurred most often between 00:00 and 4:00 and least often between 12:00 and 16:00.^63^

A multicenter study investigating the temporal changes in circadian rhythmicity in relation to disease course in 175 patients with cluster patients found that the pattern of circadian rhythmicity can change in association with disease progression, as nighttime attacks were predominant early in the disease course, while daytime attacks increased with disease progression and decreased in patients with the most advanced disease course.^64^

A large study was conducted by Steinberg et al., in a cohort of 475 patients, of which 421 experienced episodic cluster headaches and 54 experienced chronic cluster headaches. The most commonly reported time interval for attack was nighttime (2:00–4:00), and the lowest rates were noted around late morning and early afternoon (10:00–14:00). A third of patients noted no rhythmicity.^65^

In summary, cluster headache pain and frequency appear to be highest at night, and lowest in early afternoon.

#### Cancer pain

A 2019 article found that chronobiology plays a prominent role in the circadian physiology of cancer, through genes that control potentially painful oscillation processes.^66^ A prospective cohort carried out in 123 cancer patients, identified that breakthrough pain had a peak at 10:00 (*p* < 0.001). In cancer patients on chronic opioid therapy, a similar pattern was seen, with patients experiencing more breakthrough pain in the morning hours when compared to the evening. For further analysis, cancer patients were split into two subgroups, based on whether they had visceral or bony metastasis and the circadian nature of pain in both groups was characterized separately. In patients with bony metastases, the acrophase was found to be at 9:45 (*p* < 0.0001) and in patients with visceral metastasis the acrophase was at 10:30 (*p* < 0.001).^67^

In a more recent study, cosinor analysis showed a similar circadian rhythmic pattern of BTP episodes. Ninety‐two subjects with life expectancies of less than 120 days were analyzed (44 in home care, 48 in hospice). The acrophase of all patients was 12:30 (*p* < 0.001). For patients with bone metastases, acrophase was 12:15 (*p* < 0.001) and patients with visceral metastases acrophase was 12:30. For patients with an Eastern Cooperative Oncology Group (ECOG) performance score of 3 or higher, acrophase was at 13:00 (*p* < 0.01). For patients with an ECOG score of less than 2, acrophase was at 12:15.^68^ It should be noted that this study exhibited a risk of both selection and performance bias.

Patterns of breakthrough pain in cancer patients with delirium were found to be different. In a retrospective analysis of data collected for prospective study, circadian rhythm of breakthrough analgesia was different in 104 patients with or without delirium. Patients without delirium needed more analgesia in the morning (*p* < 0.001) and patients with delirium required more analgesia in the evening and at night (*p* = 0.02).^69^ It should be noted that this study exhibited a risk of selection bias.

In a randomized, double‐blind, crossover, placebo‐controlled study of 42 patients with advanced cancer, the sustained release of a once‐daily morphine sulfate (MS) dose in the morning versus evening had been studied.^70^ The results of this study showed no statistical significant difference between two groups. The mean of pain based on a 100 mm visual analogue scale (VAS) was 16 mm for the patients with MS doses in the morning and 14 mm for the patients with doses in the evening (*p* = 0.34).^70^

A much earlier study from 1976 showed intractable pain due to cancer to linearly increase throughout the day with maximum pain experienced at 22:00. Forty‐one patients (17 females) reported their pain every 2 h from 8:00 to 22:00, on a VAS for 7 consecutive days.^71^

To summarize, cancer pain and breakthrough pain do not show a clear circadian rhythmicity based on current available evidence.

## DISCUSSION

The aim of this review was to synthesize circadian pain patterns of human pain conditions. As a result, postoperative pain, fibromyalgia, trigeminal neuralgia, and migraines showed higher pain scores and episode frequency in the morning. In the afternoon, tension headaches could be expected in higher frequency. Temporomandibular joint pain and neuropathic pain were typically worst in the evening. At night, labor pain, biliary colic pain, and cluster headaches exhibited higher pain scores and episodic frequency. Furthermore, the circadian rhythm of pain was not found to be altered in patients on analgesics. On the other hand, different forms of arthritis and cancer pain were not associated with any circadian rhythms.

It must be noted that the studies included here are largely observational, many of them several decades old. The risk of bias assessment aims to qualify how studies address some intrinsic limitations of such studies, such as uniformity of the study population, and efforts to obtain natural measurements at all times of day. Nonetheless, the majority of included studies did not exhibit a significant risk of bias, and there was very little variability between the patterns identified in different studies, increasing confidence in the results of this review.

### Factors affecting circadian variation of pain

Several recent reviews have been published concerning mechanisms of circadian variation of pain, such as the one by Bumgarner et al.^72^ Of note, Warfield et al.^73^ and Tanaka et al.^74^ discuss circadian mechanisms affecting chronic pain. Similarly, Kim et al.^75^ and Hu et al.^76^ provide in‐depth discussions of circadian mechanisms affecting neuropathic pain.

In the following discussion, we touch on a few additional circadian mechanisms of pain that have been studied, mostly in animals. As expected, the trends in the studies below do follow the circadian trends in the rest of the review.^77^ It is also worth noting, the description and depth of mechanisms to follow are intentionally left short, as describing mechanisms are beyond the scope and intent of this systematic review.

To evaluate clinical factors affecting the circadian variation of pain, data from two previous double‐dummy crossover clinical trials conducted in patients with neuropathic pain were pooled together. In both of the trials, which were described before, patients were asked to rate their pain three times daily at 08:00, 16:00 and 20:00 starting from 7 days before the trial until the end of the trial. The degree of diurnal variation was defined as a difference in pain intensity between 08:00 and 20:00 averaged over 7 days. The baseline data from the preclinical time period were subjected to bivariable analysis order to find out the effect of sex, age, etiology, severity of short‐form McGill pain questionnaire (SF‐MPQ) pain quality and allodynia with respect to degree of diurnal variation. Among the many clinical factors only sex and etiology were found to be significant enough to be included in a multivariable analysis. The diurnal variation of pain was more prominent in females (*p* = 0.03) when compared to males, and the variation was also more prominent in DN when compared to PHN (*p* = 0.03).^50^

### Circadian variation of pain, Enkephalins and endorphins

Enkephalins and endorphins play an important role in the perception of pain.^78^, ^79^ Preclinical studies done on mice have shown that circadian variations in pain can be correlated to the level of endogenous opioids.^79^ In experimental studies on mice kept in a light dark cycle of 12 h (light on 06:00), the levels of metenkephalin in the entire brain were found to follow a circadian rhythm. Metenkephalin levels were twice as high at the end of resting phase when compared to the beginning of the resting phase.^80^

Circadian variation also applied to the level of beta‐endorphins present in the pituitary gland, pons, medulla, and the cerebellum of rat brain, with a peak occurring at the beginning of the activity phase in the mice.^81^ Besides endogenous opioids, changes in serotonin have been shown to influence pain sensitivity in rats.^82^

Other studies have also shown that beta‐endorphins peak and dip with activity phases in mice altering serotonin and pain sensitivities. A study performed using a murine tibia fracture model determined that nonsteroidal anti‐inflammatory drugs (NSAIDs) are most effective in managing postoperative pain, healing and recovery when drug administration is limited to the active phase of the circadian rhythm. Several genes involved in the circadian clock pathway are upregulated, such as Per2, Nr1d1 and Nr1d2 were upregulated in mice who received NSAIDs during the active phase, compared to the resting phase.^81^, ^82^

A study on opioid secretion in humans was conducted where blood was collected from seven male volunteers via a polyethylene tube inserted between lumbar vertebrae. Plasma was sampled for endogenous opioids, demonstrating a peak in the morning and nadir in the late evening (10:00, 22:00). Circadian rhythm was shown to present by analysis of variance in plasma (*p* < 0.05).^83^

A study conducted in six healthy adult volunteers showed a circadian pattern in the plasma levels of beta‐endorphin levels with a peak level at 08:00 (6.5 ± 0.5 fmol/ml) and the trough levels at 20:00 (3.7 ± 0.6 fmol/ml).^84^ A similar pattern was observed in neonates with minimal stress, neonates with severe stress and adults, where endorphin levels were high in the morning when compared to the evening. In a separate study, a highly significant variation in the levels of beta‐endorphin was demonstrated in 17 neonates where blood was drawn at 09:00, 12:00 and 15:00. The levels of beta‐endorphin were found to be high in the morning (68.3 ± 27.7 pg/ml) when compared to afternoon (45 ± 10.8) (*p* = 0.0002).^85^ These two studies demonstrate a trend in diurnal activity of beta‐endorphins, suggesting a circadian rhythm is present.

In the case of dental pain, studies have been demonstrated the presence of peripheral clocks in the production of dental pulp, periodontal tissues, oral mucosa, enamel, dentin, and mandibular bone.^86^

### Opioid receptor modulation

One group demonstrated that there is a circadian variation in opiate receptor binding in the forebrain of rats. A peak of opioid receptor binding was found at 22:00 during the activity period and a trough at 02:00.^87^ Recent studies in mice with neuropathic pain demonstrated changes in circadian rhythm of mRNA expression of mu opioid receptors. Neuropathic pain models in mice were created by performing a sciatic nerve ligation. In sham‐operated mice the latency of hind paw withdrawal response to thermal stimuli was greater at 14:00 and 20:00 when compared to that at 08:00 and 02:00, which suggested a rest period dominant circadian rhythm. But in mice which underwent sciatic nerve ligation, the latency to thermal stimuli was higher at 08:00 and 02:00 when compared to 14:00 and 20:00. The changes in latencies to thermal stimuli were found to be correlated with mRNA expression of mu opioid receptors in periaqueductal gray matter in both type of mice.^88^

One animal study used RNA sequencing profiles to investigate gene networks involved in the circadian rhythm in mice with chronic morphine exposure. The results show that circadian rhythm processes were enriched among the genes oveexpressed in mice with opioid‐induced hyperalgesia relative to control mice.^89^

### The role of substance P

The neurotransmitter substance P modulates sensitivity to pain by activating the neurokinin‐1 (NK‐1) receptor.^90^ Levels of substance P have also been shown to follow a circadian rhythm. In studies done in the substantia nigra and central gray matter of rat brain, the levels of substance P peaked between 20:00 and 04:00.^91^ The reason for the fluctuation of substance P levels was that the expression of Tac1 gene, which codes substance P, was found to oscillate under the transcription regulation of Circadian Loco motor Output Cycles Kaput (CLOCK) genes, affecting both the persistence and period of circadian rhythms.^92^

### Melatonin and pineal gland

For a recent, in‐depth review of mechanisms connecting sleep and the circadian rhythm to neuropathic and inflammatory pain, headaches, and analgesia, see Palada et al.^93^ In a study conducted in mice kept in light dark cycle, mice were treated with saline or luzindole (melatonin receptor antagonist), before subcutaneous injection of formalin to the hind paw. In mice treated with saline prior to the injection of formalin, the pain‐related behavior was heightened in the dark phase when compared to the light phase. But in mice pretreated with luzindole, the pain‐related behavior in the dark phase was similar to that of the light phase. In the same study, tonicnocicepitve responses in nocturnal hours were found to be lesser in mice with the inhibition of melatonin realease by artificial penalectomy, suggesting a proalgesic role of endogenous melatonin in tonic pain.^94^

In a study done to determine the cause of sleep disturbances in neuropathic pain, mRNA expression of melatonin receptors in the hypothalamus was found to be altered in mice with sciatic nerve ligation. The mRNA expression of the melatonin receptor was studied in sham‐operated mice and in mice which underwent sciatic nerve ligation by reverse transcription polymerase chain reaction. Sciatic nerve ligated mice which exhibited thermal hyperalgesia, increased wakefullness and decreased nonrapid eye movement (NREM) sleep at 02:00, when compared to sham operated mice.^95^

Administration of melatonin has been demonstrated to produce antinociceptive effects in acute neuropathic and inflammatory pain.^96^ For instance, a post‐marketing surveillance study was carried out to determine the efficacy and safety of melatonin supplements in 61 patients with chronic tension headaches. After 30 days of melatonin treatment, a significant decrease in number of headaches per month and VAS pain intensity were observed.^97^

## CONCLUSION

Due to pragmatic and ethical limitations, at this stage, most human studies associating pain with circadian patterns remain observational, with restricted possibility of carrying out repeatable, genuine, scalable multivariate experimentation. While it remains almost impossible to connect all circadian rhythmicity to all mechanisms for pain behaviors, there is a compelling amount of correlation between human chronobiology and pain patterns. A circadian pattern of pain was found to be present in nociceptive, neuropathic, central and mixed pain states. Postoperative pain, fibromyalgia, trigeminal neuralgia, and migraines were associated with higher pain scores in the morning. Temporomandibular joint pain, neuropathic pain, labor pain, biliary colic, and cluster headaches increased throughout the day to reach a peak in the evening or night. Arthritis and cancer pain were not associated with any circadian rhythmicity. Furthermore, the circadian rhythm of pain was not found to be altered in patients on analgesics. The results of this review suggest that there may be benefits in further studying the potential benefits of staggered dosing.

## CONFLICT OF INTEREST

The authors have no conflicts of interest to declare.

## Data Availability

All manuscripts used in this systematic review will be available from the corresponding author upon request.
